# The role of vitamin D in pregnancy outcomes in gestational diabetes: a randomized controlled trial

**DOI:** 10.1530/EC-25-0932

**Published:** 2026-02-14

**Authors:** Yifan Liu, Liying Luo, Jiani Yu, Qin Yin

**Affiliations:** ^1^ Department of Endocrinology, The Second Hospital of Lanzhou University, Lanzhou, Gansu, China; ^2^ Department of Ophthalmology, Tongren Hospital, Shanghai Jiao Tong University School of Medicine, Shanghai, China; ^3^ Department of Rheumatology and Immunology, Xuzhou Municipal Hospital Affiliated with Xuzhou Medical University, Xuzhou, Jiangsu, China; ^4^ Department of Orthopedics, Wuxi Ninth People’s Hospital Affiliated to Soochow University, Wuxi, Jiangsu, China

**Keywords:** vitamin D, gestational diabetes mellitus, oxidative stress

## Abstract

**Background:**

This study evaluated the effects of vitamin D supplementation on oxidative stress, inflammation, and pregnancy outcomes in women with gestational diabetes mellitus (GDM).

**Methods:**

In a randomized, double-blind, placebo-controlled trial, 229 women with GDM were assigned to receive 200 IU of vitamin D twice daily or a placebo for 6 weeks alongside standard care. Biomarkers and pregnancy outcomes were assessed.

**Results:**

Vitamin D supplementation significantly increased serum 25(OH)D and calcium levels, improved oxidative stress markers (increased GSH and decreased MDA), and reduced hs-CRP compared to placebo. The vitamin D group had a lower neonatal birth weight and a reduced incidence of macrosomia. No significant differences were found in fasting glucose changes or most other obstetric outcomes.

**Conclusion:**

A 6-week vitamin D supplementation in women with GDM improved vitamin D status, ameliorated oxidative stress and inflammation, and was associated with reduced neonatal birth weight and macrosomia, without significantly affecting glycemic control.

## Introduction

Pregnancy is when gestational diabetes mellitus (GDM), a disorder of glucose intolerance, is first diagnosed (1). Key risk factors for GDM development include gestational age, obesity, ethnicity, family history of type 2 diabetes mellitus (T2DM), and prior GDM (2). GDM has long been linked to obstetric and neonatal complications – primarily due to elevated birth weight – and is increasingly recognized as a risk factor for future cardiometabolic disease in both mothers and offspring (3, 4). Global GDM prevalence continues to rise, influenced by the new International Association of Diabetes and Pregnancy Study Groups (IADPSG) diagnostic criteria and epidemiologic factors, such as increasing maternal age and obesity prevalence among women of reproductive age (5). Given GDM’s impact on pregnancy and fetal outcomes, selecting appropriate therapeutic interventions is essential.

Maternal nutritional intake during pregnancy affects newborn vitamin D deficiency risk (6). Rising maternal vitamin D deficiency may stem more from dietary and sunlight exposure changes than increased physiological demand (7, 8). Notably, women often transfer calcium to the fetus without adequate vitamin D, potentially explaining the high prevalence of vitamin D insufficiency (9). As the fetus relies entirely on maternal vitamin D, deficiency may impair fetal/child development and maternal health (10). While guidelines for increased pregnancy dietary requirements remain limited, due to insufficient evidence linking maternal 25(OH)D levels to optimal maternal–fetal skeletal outcomes (11), some observational studies suggest that maternal vitamin D benefits offspring’s skeletal development (12).

Within the complex landscape of oxidative stress in GDM, glutathione (GSH) plays a pivotal role (13). As the most abundant non-enzymatic intracellular antioxidant, GSH is fundamental for neutralizing reactive oxygen species (ROS), detoxifying xenobiotics, and maintaining the redox balance of crucial cellular proteins (14). In pregnancy, an adequate GSH reservoir is essential for protecting both maternal and fetal tissues from oxidative damage (15). However, GDM is characterized by a state of heightened oxidative stress that can deplete antioxidant reserves. Emerging evidence indicates that women with GDM exhibit significantly lower levels of GSH compared to their normoglycemic counterparts, a depletion that is correlated with the severity of insulin resistance and hyperglycemia (16, 17, 18). More critically, this compromised antioxidant defense has been directly linked to adverse clinical outcomes. Studies have demonstrated that lower maternal GSH levels are associated with an increased risk of fetal macrosomia, preterm birth, and preeclampsia, likely due to unchecked oxidative damage impacting placental function and fetal development. Furthermore, the postnatal sequelae of this oxidative environment, including neonatal metabolic disturbances, may also be influenced by maternal antioxidant status (19, 20). Therefore, investigating interventions that can bolster the GSH system is of clear clinical importance. Given vitamin D’s reported role in modulating antioxidant gene expression and reducing oxidative stress, we hypothesized that vitamin D supplementation could positively influence GSH levels, thereby potentially mitigating one of the key pathological pathways in GDM and improving associated clinical endpoints.

Given the limited evidence from interventional trials supporting vitamin D as a therapeutic agent for gestational diabetes, we conducted this randomized controlled trial to investigate the specific effects of a 6-week vitamin D supplementation regimen in women with GDM. The primary outcome was the change from baseline in biomarkers of oxidative stress (glutathione, GSH; malondialdehyde, MDA) and inflammation (high-sensitivity C-reactive protein, hs-CRP). Secondary outcomes included changes in maternal serum 25-hydroxyvitamin D and calcium levels, measures of glycemic control (fasting and postprandial glucose), and a range of pregnancy and neonatal outcomes (including birth weight, macrosomia, mode of delivery, and preterm birth) (21, 22).

## Materials and methods

This section has been revised in accordance with the Consolidated Standards of Reporting Trials (CONSORT) guidelines to ensure comprehensive and transparent reporting of trial methodology (Supplementary Materials, see section on Supplementary materials given at the end of this article).

### Trial design and setting

This study was a single-center, prospective, randomized, double-blind, placebo-controlled, parallel-group trial. It was conducted at a tertiary care hospital from January 2020 to December 2022. The study protocol was approved by the Hospital’s Ethics Committee and Institutional Review Board (IRB) (Approval No. SHTR-2015-58). The trial was conducted in accordance with the ethical principles of the Declaration of Helsinki. All participants provided written informed consent before enrollment.

### Participants

Pregnant women aged 18–40 years diagnosed with GDM between 24 and 28 weeks of gestation were eligible. GDM was diagnosed using a one-step 75 g oral glucose tolerance test (OGTT) according to American Diabetes Association criteria: fasting plasma glucose (FPG) ≥ 92 mg/dL, 1 h plasma glucose ≥ 180 mg/dL, or 2 h plasma glucose ≥ 153 mg/dL.

Exclusion criteria included the following: prior use of additional magnesium, zinc, calcium, or vitamin D supplements beyond the standard prenatal care protocol (which included a daily multivitamin containing 1,000 IU vitamin D); smoking; insulin therapy initiated post-GDM diagnosis; pre-eclampsia; eclampsia; hypo-/hyperthyroidism; significant renal or hepatic disorders; and placental abruption.

Participants were randomly allocated in a 1:1 ratio to the vitamin D or placebo group using a computer-generated random number sequence. The allocation sequence was concealed from the investigators and participants using sequentially numbered, opaque, sealed envelopes prepared by an independent statistician not involved in the trial. The study was double-blind: the participants, healthcare providers, outcome assessors, and data analysts were all blinded to group assignment until the final statistical analysis was completed.

### Allocation of treatments and randomization

Eligible participants were randomly assigned to one of the two groups for a 6-week intervention period: i) The intervention group received oral supplements containing 200 IU of vitamin D (cholecalciferol), taken twice daily (total daily dose: 400 IU) and ii) the control group received an identically appearing and tasting placebo, administered on the same twice-daily schedule.

Per standard Iranian prenatal care guidelines, all enrolled participants were taking a daily multivitamin containing 1,000 IU vitamin D and 400 μg folic acid from conception, plus 60 mg ferrous sulfate daily from the second trimester. The study intervention (200 IU vitamin D or placebo twice daily) was administered in addition to this background standard care.

### Outcomes

A certified midwife performed anthropometric assessments pre- and post-intervention. Weight and height were measured in light clothing without shoes using a Seca 713 scale (nearest 0.1 kg and 0.1 cm, respectively). BMI was calculated as weight in kilograms divided by the square of height in meters (kg/m^2^). Post-delivery, infant weight and length were measured using standardized methods (Seca 155 Scale, Hamburg, Germany), and head circumference was recorded to the nearest 1 mm with a Seca tape. Neonatal 1- and 5-min Apgar scores were documented.

Polyhydramnios (excess amniotic fluid) was identified via ultrasound at trial conclusion, defined as an amniotic fluid index > 25 cm. Preterm delivery was defined as birth at <37 weeks of gestation. Neonatal macrosomia was defined as a birth weight >4,000 g; large-for-gestational-age (LGA) was defined as a birth weight >90th percentile for sex/gestational age (23, 24).

Ten milliliters of fasting blood were drawn for biomarker tests at weeks 0 and 6 of the intervention. Enzyme kits with intra- and inter-batch coefficient variation (CV) less than 4.2% were used to quantify serum calcium concentrations. An enzymatic kit was used to calculate FPG. The blood 25-hydroxyvitamin D concentration was measured using an ELISA kit (IDS, UK). The respective CV rates for the batches were 4.65 and 6.98%. An ELISA kit (LDN, Germany) was used to determine the levels of serum hs-CRP. The intra-batch and inter-batch CVs were 3.26 and 7.25%, respectively. An ELISA kit (LDN, Germany) was used to determine the levels of serum hs-CRP. The intra-batch and inter-batch CVs were 3.26 and 7.25%, respectively. After part of the blood sample was centrifuged (3,000 *g*, 10 min, 4°C) for collection, the plasma was separated. Once the assessments of total nitrite, malondialdehyde (MDA), total antioxidant capacity (TAC), and GSH were finished (25), it was kept at −70°C. The concentration of GSH19 was measured using the Beutler *et al.* technique, whereas the concentration of plasma total nitrite was measured using the Griess method (26). MDA concentrations were determined spectrophotometrically using compounds that react with thiobarbituric acid (27). Benzie and Strain’s iron-reducing antioxidant capacity technique was used to assess the amount of TAC in plasma (28). TAC, MDA, GSH, and total plasma nitrite all had CVs below 4.25%. Serum total bilirubin levels more than 15 mg/dL (257 μmol/L) in infants aged 25–48 h, larger than 18 mg/dL (308 μmol/L) in infants aged 49–72 h, and greater than 20 mg/dL (342 μmol/L) in infants older than 72 h are considered neonatal hyperbilirubinemia (29).

### Estimating the sample size

The sample size was determined *a priori* for the primary outcome, which was the change in glutathione (GSH) levels. Using G*Power software (version 3.1.9.7) (30), a two-tailed, two-independent-means *t*-test was specified. Based on previous data from a similar population investigating micronutrient supplementation on oxidative stress markers, we estimated a standardized effect size (Cohen’s d) of 0.45. To achieve 80% power at a significance level (α) of 0.05, a minimum of 78 participants per group was required. Anticipating a potential attrition rate of approximately 15% over the 6-week intervention period, we aimed to recruit 92 participants per group. To accommodate practical enrollment and ensure adequate power, a final target of 229 participants (124 in the vitamin D group; 105 in the placebo group) was set (31, 32, 33, 34, 35).

### Predictive nomogram evaluation

LASSO regression identified sepsis-induced acute hepatic dysfunction risk factors after multivariate data reduction. Non-zero LASSO coefficients were applied to the training set. Significant LASSO-selected variables underwent multiple logistic regression to build a predictive model. The nomogram’s discriminative power was evaluated via Harrell’s C-index, calibration via calibration curves, and clinical utility via decision curve analysis. Model performance was validated using ROC curves (AUC) (36).

### Statistical analyses

Normality was assessed using Kolmogorov–Smirnov tests (all variables non-parametric, *P* < 0.05). Continuous variables (pre-pregnancy BMI, maternal weight gain, OGTT results, maternal age, gestational age, and birth weight) were compared using Mann–Whitney tests. Categorical variables (excessive weight gain, GDM, parity, and delivery mode) were analyzed with Pearson chi-squared tests using standardized adjusted residuals. Analyses used SPSS 24.0 with *α* = 0.05 (two-tailed) and intention-to-treat principles.

## Results

### Participant flow and recruitment

Between January 2020 and December 2022, a total of 352 pregnant women were assessed for eligibility. After excluding 123 individuals who did not meet the inclusion criteria (*n* = 95) or declined to participate (*n* = 28), 229 women diagnosed with GDM were enrolled and randomly allocated to either the vitamin D supplementation group (*n* = 124) or the placebo group (*n* = 105). All 229 participants received their allocated intervention and were included in the intention-to-treat (ITT) analysis. During the 6-week intervention period, there were five losses to follow-up in the vitamin D group (4%) and four in the placebo group (3.8%), primarily due to relocation or withdrawal of consent. Data from these participants were carried forward using the last observation carried forward (LOCF) method for the analysis of biomarker changes. All participants were followed for pregnancy outcomes until delivery.

### Baseline characteristics

Baseline demographic and clinical characteristics of the participants are presented in Table 1. The two groups were well-balanced at enrollment, with no statistically significant differences in age, educational background, parity, family history of diabetes, gestational age, anthropometric measures (height, pre-pregnancy weight, and BMI), glycemic indices at GDM diagnosis (OGTT values), or pre-intervention fasting and postprandial glucose levels (all *P* > 0.05).

**Table 1:** Comparison of general characteristics of patients with GDM between the two groups.

Characteristic	Placebo group (105)	Vitamin D supplement group (124)	Test value	*P* value
Age (years, mean ± SD)	30.25 ± 4.89	30.99 ± 4.85	0.28	0.77
Education (*n*, %)				
Primary school	6	8		
Junior high school	8	8		
Technical secondary school to high school	10	9		
College and above	65	60		
Parity number (parity, M, IQR)	2 (1–3)	2 (1–4)	0.68	0.11
Family history of diabetes (*n*, %)	26	29	1.58	0.38
History of cesarean section (*n*, %)	28	32	2.61	0.27
Current gestational age (weeks, mean ± SD)	29.35 ± 3.65	28.95 ± 4.84	1.99	0.54
Height (m, mean ± SD)	1.58 ± 0.21	1.59 ± 0.34	0.58	0.41
Weight before pregnancy (kg, mean ± SD)	59.64 ± 11.25	58.99 ± 12.54	1.25	0.62
BMI before pregnancy (kg/m^2^, mean ± SD)	23.68 ± 5.14	23.11 ± 2.61	2.15	0.55
Gestational age at diagnosis of GDM (weeks, mean ± SD)	25.36 ± 1.08	25.68 ± 1.11	1.28	0.42
Weight at diagnosis of GDM (kg, mean ± SD)	66.25 ± 4.25	66.39 ± 3.28	2.61	0.85
OGTT: fasting blood glucose (mmol/L, mean ± SD)	5.17 ± 0.05	5.12 ± 0.24	0.98	0.22
OGTT: 1 h postprandial blood glucose (mmol/L, mean ± SD)	10.25 ± 0.62	10.15 ± 1.25	0.24	0.64
OGTT: 2 h postprandial blood glucose (mmol/L, mean ± SD)	8.26 ± 1.15	8.62 ± 1.05	0.39	0.44
Pre-intervention fasting blood glucose (mmol/L, mean ± SD)	5.66 ± 0.85	5.39 ± 1.25	1.28	0.62
Pre-intervention 2 h postprandial blood glucose (mmol/L, mean ± SD)	6.35 ± 0.85	6.65 ± 1.25	0.85	0.25

### Effects on biochemical parameters

Changes in biochemical markers from baseline to the end of the 6-week intervention are detailed in Table 2. Post-intervention, the vitamin D group exhibited a significantly greater increase in serum 25-hydroxyvitamin D levels compared to the placebo group (mean change: 6.38 ± 3.51 ng/mL vs 4.25 ± 2.68 ng/mL; *P* = 0.021). Similarly, the increase in serum calcium was more pronounced in the intervention group (0.86 ± 0.09 mg/dL vs 0.25 ± 0.02 mg/dL; *P* = 0.001). Regarding oxidative stress and inflammation, vitamin D supplementation led to a significant rise in glutathione (GSH) (22.15 ± 3.21 μmol/L vs −12.65 ± 6.28 μmol/L; *P* = 0.001) and a significant reduction in malondialdehyde (MDA) (−0.89 ± 0.03 μmol/L vs 0.34 ± 0.08 μmol/L; *P* = 0.003) and high-sensitivity C-reactive protein (hs-CRP) (−1.28 ± 0.25 mg/L vs 0.95 ± 0.12 mg/L; *P* = 0.019) compared to placebo. No significant between-group differences were observed in the changes of fasting blood glucose, total nitrite, or total antioxidant capacity (TAC) (all *P* > 0.05).

**Table 2:** Comparison of post-intervention indicators of patients with GDM between the two groups.

Characteristic	Placebo group (*n* = 105)			Vitamin D supplement group (124)			*P* value
	0 weeks	6 weeks	Change	0 weeks	6 weeks	Change	
FPG (mg/dL)	95.62 ± 5.26	93.25 ± 2.68	&-0.95 ± 0.15	93.12 ± 1.28	82.61 ± 2.64	&-5.62 ± 1.05	0.015
Calcium (mg/dL)	9.12 ± 0.45	9.15 ± 0.28	0.25 ± 0.02	9.24 ± 0.24	9.56 ± 0.32	0.86 ± 0.09	0.001
25(OH)D (ng/mL)	13.58 ± 2.18	17.39 ± 1.58	4.25 ± 2.68	13.64 ± 1.35	18.62 ± 2.68	6.38 ± 3.51	0.021
hs-CRP (mg/L)	6.38 ± 0.89	7.98 ± 2.89	0.95 ± 0.12	6.23 ± 0.99	5.02 ± 1.02	&-1.28 ± 0.25	0.019
Total nitrite (μmol/L)	40.28 ± 12.08	41.89 ± 8.95	0.93 ± 0.11	40.58 ± 8.64	44.58 ± 6.51	2.68 ± 5.94	0.126
TAC (mmol/L)	698.65 ± 58.69	664.28 ± 89.95	&-25.61 ± 8.25	699.64 ± 86.15	725.64 ± 95.64	29.64 ± 5.69	0.065
GSH (μmol/L)	477.62 ± 85.61	461.05 ± 85.62	&-12.65 ± 6.28	471.26 ± 75.61	498.65 ± 96.45	22.15 ± 3.21	0.001
MDA (μmol/L)	3.21 ± 0.78	3.56 ± 0.95	0.34 ± 0.08	3.26 ± 0.85	2.95 ± 0.08	&-0.89 ± 0.03	0.003
Mean fasting glucose (mmol/L)	5.66 ± 0.85	5.69 ± 0.66	0.031 ± 0.002	5.39 ± 1.25	5.62 ± 0.26	0.26 ± 0.11	0.06
Mean 2 h postprandial blood glucose	6.35 ± 0.85	6.68 ± 0.32	0.32 ± 0.15	6.65 ± 1.25	6.89 ± 0.64	0.25 ± 0.09	0.28

### Pregnancy and neonatal outcomes

Key maternal and neonatal outcomes are summarized in Table 3. The incidence of neonatal macrosomia (birth weight >4,000 g) was significantly lower in the vitamin D group (4%, 5/124) than in the placebo group (17%, 18/105) (*P* = 0.02). Correspondingly, the mean neonatal birth weight was significantly lower in the vitamin D supplementation group (3,014.23 ± 445.21 g) compared to the control group (3,348.2 ± 421.68 g) (*P* = 0.03). No statistically significant differences were found between the two groups regarding the rates of cesarean section, preterm delivery, pre-eclampsia, polyhydramnios, neonatal hyperbilirubinemia, hospitalization, or Apgar scores at 1 and 5 min (all *P* > 0.05).

**Table 3:** Association between vitamin D supplement and newborn outcomes.

Characteristic	Placebo group (105)	Vitamin D supplement group (124)	*P* value
Cesarean section (%)	25	12	0.26
Preterm delivery (%)	4	1	0.32
Need to insulin therapy after intervention (%)	6	3	0.25
Pre-eclampsia (%)	10	7	0.62
Polyhydramnios (%)	6	4	0.05
Macrosomia > 4,000 g (%)	17	4	0.02
Newborns’ weight (g)	3,348.2 ± 421.68	3,014.23 ± 445.21	0.03
Newborns’ length (cm)	52.64 ± 2.61	49.25 ± 1.15	0.48
Newborns’ head circumference (cm)	35.26 ± 1.54	34.05 ± 2.61	0.62
1 min Apgar score	8.95 ± 0.48	5.89 ± 0.44	0.28
5 min Apgar score	9.58 ± 0.32	9.95 ± 0.33	0.32
Newborns’ hyperbilirubinemia (%)	42	34	0.28
Newborns’ hospitalization (%)	40	32	0.48

### Regression and predictive model analysis

To identify factors associated with the response to vitamin D supplementation, univariate and multivariate analyses were performed (Table 4). Univariate analysis indicated significant associations with fasting glucose, 25-hydroxyvitamin D, and GSH levels. In the subsequent multivariate logistic regression model, only 25-hydroxyvitamin D (HR: 1.02, 95% CI: 0.98–1.96; *P* = 0.02) and GSH (HR: 0.98, 95% CI: 1.11–0.48; *P* = 0.01) remained as independent predictive factors.

**Table 4:** Univariate and multivariate Cox proportional hazards regression model.

	Univariate analysis			Multivariate analysis		
	HR	95% CI	*P* value	HR	95% CI	*P* value
Newborns’ weight (g)	1.25	0.59 (0.44–1.45)	0.06	0.97	1.28 (1.05–2.65)	0.08
OGTT: fasting blood glucose	0.89	0.98 (0.75–1.11)	0.02	2.11	2.05 (0.89–3.26)	0.51
25(OH)D	0.88	0.68 (0.24–1.15)	0.01	1.02	0.98 (0.58–1.96)	0.02
Polyhydramnios	1.69	0.38 (0.11–1.21)	0.65	2.36	2.22 (1.29–3.99)	0.07
GSH	0.96	0.67 (0.33–1.95)	0.02	0.98	1.11 (0.48–1.88)	0.01
TAC	0.86	0.96 (0.77–1.85)	0.34	0.77	1.68 (0.49–1.95)	0.15

**Figure 1:**
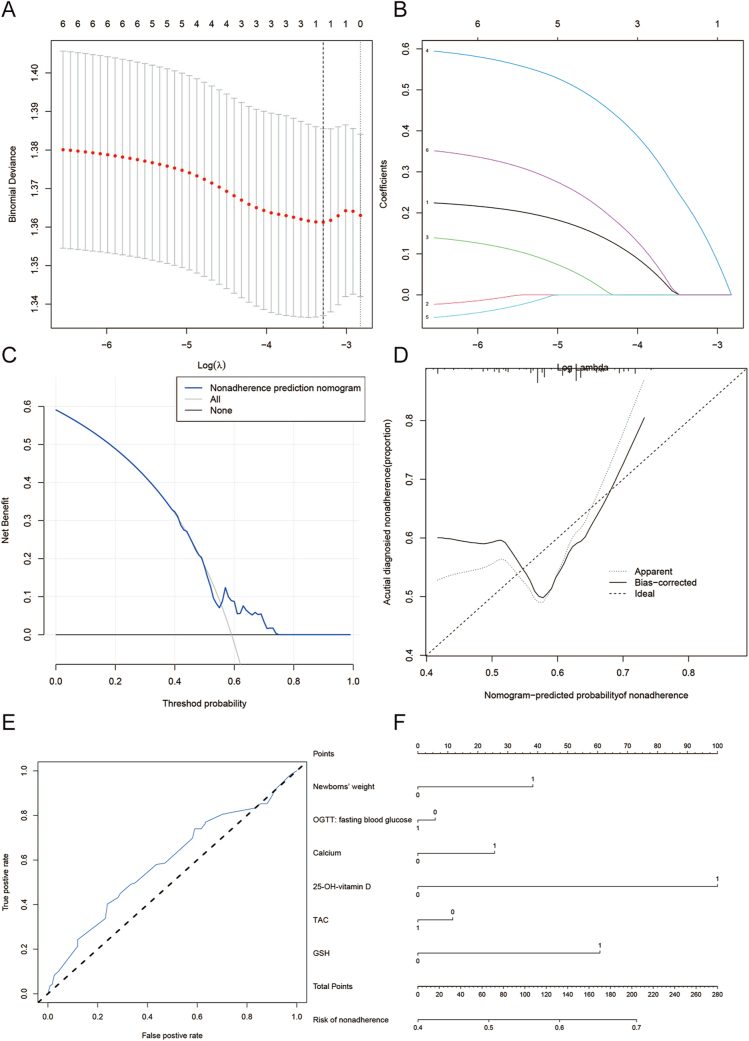
LASSO regression analysis between the two groups.

Furthermore, a least absolute shrinkage and selection operator (LASSO) regression model was applied to select the most relevant predictive variables from 25 initial candidates. The model identified five variables with non-zero coefficients: calcium, GSH, TAC, 25-hydroxyvitamin D, and fasting OGTT value. These were incorporated into a prognostic nomogram (Fig. 1). The model demonstrated excellent discriminative ability, with a C-index of 0.948 (95% CI: 0.045–1.385), which was validated as 0.955 through bootstrapping. The area under the receiver operating characteristic curve (AUC) was 0.995. Decision curve analysis confirmed the clinical utility of the model across a wide range of threshold probabilities.

## Discussion

GDM refers to impaired glucose tolerance first diagnosed during pregnancy (37, 38, 39). While historically associated with perinatal complications (particularly fetal macrosomia), GDM is increasingly recognized as a risk factor for future cardiometabolic disease in both mothers and offspring (40, 41, 42). In our study, vitamin D intervention significantly modulated oxidative stress markers and elevated calcium/vitamin D levels in pregnant women with diabetes. It also reduced neonatal birth weight, with regression models identifying vitamin D and total antioxidant capacity (TAC) variations as primary risk factors.

Vitamin D (VitD) levels strongly correlate with numerous metabolic functions, including immune regulation, cellular proliferation, and glucose homeostasis (43, 44, 45). Emerging evidence suggests that vitamin D influences metabolic profiles in menopausal women and critically impacts reproductive outcomes (46). Our results confirm that vitamin D supplementation significantly increases maternal calcium and vitamin D concentrations. This meta-analysis (*n* = 2,146) linking lower 25(OH)D to GDM risk (OR 1.61; 95% CI: 1.19–2.17) (47) aligns with Aghajafari *et al.*’s review of 31 studies associating low 25(OH)D with GDM incidence (48, 49). Wei *et al.* further demonstrated that maternal 25(OH)D < 50 nmol/L increases GDM risk (OR 1.38; 95% CI: 1.12–1.70) (21). Adequate vitamin D may reduce risks of preterm birth, being SGA, preeclampsia, and GDM (50), while decreasing offspring’s susceptibility to neurodevelopmental disorders and enamel defects (51). Conversely, deficiency correlates with reduced offspring bone mineral content, ADHD, and enamel abnormalities (52, 53, 54). Collectively, vitamin D therapy effectively addresses calcium/vitamin D deficits common in GDM.

Oxidative stress and inflammation are interrelated: ROS activate inflammatory cells, increasing inflammatory mediators, while inflammation further stimulates ROS production – creating a pathogenic cycle potentially mediated by NF-κB signaling (55). GDM is characterized by elevated oxidative stress, altered TAC, and pro-inflammatory profiles (56), which may contribute to postpartum insulin resistance and cardiovascular risk. Notably, these abnormalities during pregnancy may lead to postpartum insulin resistance and increase the risk of cardiovascular disease (56, 57, 58). In particular, GDM exhibits impaired antioxidant defenses with elevated MDA, TBARS, LOOH, and xanthine oxidase (XO), alongside reduced TAC (59). Women with GDM show higher methylglyoxal (MGO), TNF-α, IL-8, and CRP levels but lower SOD and GPX activity versus controls (60, 61, 62, 63, 64, 65, 66, 67, 68). Placental tissue in GDM demonstrates increased protein carbonyls, 4-HNE, MDA, and XOD (69). When placental antioxidant capacity is overwhelmed, systemic oxidative damage may occur (70, 71), explaining the elevated oxidative stress markers observed in maternal circulation (72).

Vitamin D supplementation also has a significant effect on babies. Vitamin D levels in neonates are often low, especially in temperate areas. There is evidence that maternal vitamin D deficiency is a substantial risk factor since newborns exclusively get vitamin D from their mothers (73). Pregnant women who use vitamin D supplements have much higher serum 25(OH)D levels, which often leads to higher cord serum 25(OH)D values (74). In addition, there seems to be a positive relationship between the levels of 25(OH)D in mothers and newborns, the advice of medical professionals about vitamin D, and mothers’ adherence to supplementation (6). However, in the winter, the risk of maternal–fetal transmission of vitamin D is reduced in the presence of obesity, low socioeconomic status, lifestyle factors (e.g., smoking), and drug abuse (75). In preterm neonates, oral vitamin D supplementation has a dose- and time-dependent effect on the fraction of Treg (76). Prenatal to postpartum vitamin D3 supplementation is a helpful way to raise a mother’s vitamin D levels and promote optimal vitamin D status in newborns and exclusively breastfed infants.

Notably, a cross-sectional study from Greece, a region with abundant sunshine, specifically investigated maternal vitamin D status and neonatal anthropometrics, revealing a positive association between maternal vitamin D deficiency and lower birth weight among women receiving prenatal vitamin D supplementation, although no correlation was found with neonatal height or head circumference. This finding, albeit from a different population context, lends support to the broader significance of maternal vitamin D status in influencing fetal growth metrics, such as birth weight, an outcome also modulated in our trial through vitamin D intervention (77).

## Conclusion

According to our findings, pregnant women’s levels of oxidative stress and vitamin D are significantly raised by vitamin D supplementation. Future research will look more closely at the precise processes and potential impacts of vitamin D supplementation on gestational diabetes.

## Supplementary materials



## Declaration of interest

The authors declare no conflict of interest. The funders had no role in the design of the study; in the collection, analyses, or interpretation of data; in the writing of the manuscript, or in the decision to publish the results.

## Funding

This work was funded by Gansu Provincial Science and Technology Plan Project (Natural Science Foundation) (No. 25JRRA609) and Project of Beijing Weiai Public Welfare Foundation: The Impact of Ganagliflozin Proline Tablets on Insulin Resistance and Body Mass Index in Patients with Type 2 Diabetes Mellitus.

## Author contribution statement

YFL, LYL, JY and QY conceived the study. LYL, JY and QY curated the data. LYL, JY and QY performed formal analysis. LYL, JY and QY acquired funding. LYL, JY and QY performed data validation. LYL, JY and QY were involved in visualization. LYL, JY and QY wrote the original draft of the manuscript.

## Ethics approval and consent to participate

This study was approved by the Ethics Committee of the Second Hospital of Lanzhou University and the Institutional Review Board (IRB) (SHTR-2015-58).

## Consent for publication

All authors agreed to publication.

## Consent to participate

All patients provided consent to participate in this study.
